# Clinical Lecture on Pneumothorax

**Published:** 1896-04-18

**Authors:** S. H. Habershon

**Affiliations:** Assistant Physician and Pathologist to the Brompton Hospital for Consumption and Diseases of the Chest


### Medical Progress and Hospital Clinics.

[*The Editor will be glad to receive offers of co-operation and contributions from members of the profession. All letters should be addressed to* The Editor, at the Office, 428, Strand, London, W.C.]

### CLINICAL LECTURE ON PNEUMOTHORAX.

By S. H. Habershon, M.D., F.R.C.P., Assistant Physician and Pathologist to the Brompton Hospital for Consumption and Diseases of the Chest.

### II.—Symptoms of Pneumothorax.

**Symptoms of the Onset.**—As a rule the onset is accompanied by sudden and severe symptoms. When, previously, the disease of the affected lung has not been extensive it is easy to conceive that the sudden collapse of a comparatively healthy lung will be associated with severe shock and with extreme mechanical interference with the circulation and respiratory power. The case is different when pneumothorax supervenes through the perforation of the pleura of a lung already useless from extensive disease. In such cases the onset cannot always be definitely marked, unless the patient has been under close observation. At least, from the history that a patient gives it is not always possible to define the onset. On the other hand, if one lung is already seriously diseased and pneumothorax supervenes in the more healthy lung, death may be sudden, or the patient may live for a few hours only. Pneumothorax has been given as one of the causes of sudden death. I believe that this has been mentioned by Fagge, and when pneumothorax has occurred in the comparatively healthy lung, the other being extensively diseased, it is certainly cited by many authorities as one of the causes of sudden death. Further, the symptoms may be modified by the presence or absence of extravasation from the contents of a cavity in the lung into the pleural cavity. This has special reference to the amount of pain.

Frequently, and indeed, in most cases, pneumothorax is actually induced by a fit of violent coughing, or by a sudden muscular strain, such as exertion or straining at stool. The patient is seized with faintness and collapse, the radial pulse becomes small and frequent, or even absent, the lips blue, the extremities livid and cold, and there is urgent dyspnœa, and more or less pain in the affected side. In many cases the symptoms of collapse are followed by a rise of temperature, but in fatal cases the temperature may fall until death. A prominent symptom of the onset in many tubercular cases is a severe pain of a stabbing or lancinating character in the affected side. This pain is not always a marked symptom, and when present is due to irritation of the pleura and subsequent pleurisy. It is not uncommon for the patient to complain of a sensation of something in the chest having given way or ruptured. The respiratory movements are always accelerated, and in many cases there is a sense of constriction of the chest associated with the dyspnœa.

The temperature, as has been said, sometimes falls in cases where the collapse is followed by increasing and fatal exhaustion. The notes are shown of a case in which the temperature fell continuously in the course of the forty-eight hours preceding death from degrees at the time of the onset of the symptoms to 96·4 degrees on the day of dcath. More often, however, a considerable rise of temperature will be observed. In a few cases where there has previously been pus or serous effusion in the pleural cavity this fluid has been discharged from the mouth through one of the bronchial tubes.

The subsequent course of the symptoms and the prognosis depends upon the cause of the pneumothorax, or, in other words, upon the condition of the lungs and pleura previous to the accident of the perforation. No less than 90 per cent. of all the cases of pneumothorax occurring are said to be tubercular, and I shall, therefore, speak more fully upon this cause, especially as it is the one with which I have more acquaintance. As will be seen from the pathological specimens already shown, pneumothorax in tubercular disease may result in three classes of cases, viz.:—

1. Those cases in which the disease is extremely localised and confined to one lung, and consists of the perforation of the visceral pleura overlying a limited necrotic area of tubercle in a superficial portion of the lung. In two specimens shown a caseous area is seen. In such cases either serous or purulent effusion will probably follow, and after the symptoms of shock have passed off the case may not infrequently assume a very chronic aspect. Two of the patients to be shown to-day are of this type, and the comparatively favourable condition is probably due to the partial arrest of the disease, which in one of the cases remains confined to the lung originally affected. At the same time, two morbid specimens shown illustrate the fact that death often results from the original shock, or from the exhaustion consequent upon prolonged suppuration.

2. Cases in which there is extensive disease of one lung, while no signs are detected in the opposite lung. In such the occurrence of pneumothorax does not produce such profound shock as when the lung has previously been fairly healthy, and, indeed, the symptom need not invariably be regarded as unfavourable. The diminution in the circulation of the now compressed lung tends to still further arrest active necrosis, and such cases may become extremely chronic. They may, however, lead to a fatal issue from prolonged suppuration and analoid disease, or from extension of the disease to the opposite lung.

3. The common class of tubercular disease that occurs in this hospital is that of acute disease, extensive in one lung, but also infiltrating to a less extent one or more lobes of the opposing lung. In these the course of the disease is usually of extreme gravity, and it is generally regarded as of fatal significance. This, however, is not always so, especially when there is only incipient disease of the opposite lung. In rare cases the disease may become entirely arrested, and, without the occurrence of effusion, the aperture in the pleura become entirely sealed up and the air absorbed, while the surfaces of pleura previously separated by air become completely adherent. One such case has occurred to me quite recently, and forms a remarkable exception to the rule. Another case, originally of extreme acuteness, is shown this afternoon, in which the disease has become chronic and the condition very much improved. From the above remarks it will be seen that the progression in tubercular disease depends greatly on the extent of the lung involved, and upon the distribution of the tubercular affection. The patients shown illustrate remarkably the various treatments adopted.

**Treatment to be Adopted.**—The rules usually adopted in this hospital may be briefly stated as follows:—

1. If possible do not interfere by paracentesis. If the opening is large the withdrawal of air will be followed by an immediate re-collection. If the fluid be drawn off, even though the opening of the pneumothorax may be closed, it is common to find an access of acute tubercular disease or acute softening in the lung in which the circulation is now partially restored, and upon which the compression is relieved.

2. The circumstances under which paracentesis must be performed are: (*a*) When there is danger to life from the extreme accumulation and pressure of air, or from the embarrassment caused by a large collection of fluid; and (*b*) if there is a pneumothorax, and the patient is suffering from the effects of exhaustion or from the suppuration. A purulent effusion may, however, be left for a long period without detriment to the patient so great as that produced by its removal.

3. Aspiration introduces an increased risk of local interstitial emphysema of the chest wall, and should, if possible, be avoided.

Of the forms of pneumothorax other than tubercular there is not much to be said. I have seen one case following pneumonia, and this patient recovered without any unfavourable symptoms. Of two cases reported at the Clinical Society on the 14th inst., following typhoid fever, it was considered that they were septicæmic in origin, as both were preceded by pleurisy and effusion from the rupture of a small superficial after abscess. One case died; the second recovered, pyæmic discharging pus through a bronchus. When the cause of pneumothorax is the rupture of an emphysematous bleb the case may be favourable throughout, though two specimens are shown in which a fatal result occurred, but in each there was chronic tubercular disease present.

Recurrent pneumothorax has more than once occurred in this hospital. One case, under the care of one of my colleagues, has been in the wards on several occasions, and the pneumothorax is thought to be caused by the rupture of emphysematous blebs. A second case was of great rarity, and due to hytadid cysts in the lung rupturing into the pleural cavity.

